# VEGF Promotes Malaria-Associated Acute Lung Injury in Mice

**DOI:** 10.1371/journal.ppat.1000916

**Published:** 2010-05-20

**Authors:** Sabrina Epiphanio, Marta G. Campos, Ana Pamplona, Daniel Carapau, Ana C. Pena, Ricardo Ataíde, Carla A. A. Monteiro, Nuno Félix, Artur Costa-Silva, Claudio R. F. Marinho, Sérgio Dias, Maria M. Mota

**Affiliations:** 1 Unidade de Malária, Instituto de Medicina Molecular, Universidade de Lisboa, Lisboa, Portugal; 2 Instituto Gulbenkian de Ciência, Oeiras, Portugal; 3 Faculdade de Medicina Veterinária de Lisboa, Universidade Técnica de Lisboa, Portugal; 4 Serviço de Anatomia Patológica, Hospital de Santa Maria e Faculdade de Medicina de Lisboa, Portugal; 5 Departamento de Parasitologia, Universidade de São Paulo, São Paulo, Brasil; 6 Angiogenesis Laboratory, Centro Investigação em Patobiologia Molecular, Instituto Português de Oncologia Francisco Gentil, Centro Regional de Oncologia de Lisboa, Lisboa, Portugal; McGill University, Canada

## Abstract

The spectrum of the clinical presentation and severity of malaria infections is broad, ranging from uncomplicated febrile illness to severe forms of disease such as cerebral malaria (CM), acute lung injury (ALI), acute respiratory distress syndrome (ARDS), pregnancy-associated malaria (PAM) or severe anemia (SA). Rodent models that mimic human CM, PAM and SA syndromes have been established. Here, we show that DBA/2 mice infected with *P. berghei* ANKA constitute a new model for malaria-associated ALI. Up to 60% of the mice showed dyspnea, airway obstruction and hypoxemia and died between days 7 and 12 post-infection. The most common pathological findings were pleural effusion, pulmonary hemorrhage and edema, consistent with increased lung vessel permeability, while the blood-brain barrier was intact. Malaria-associated ALI correlated with high levels of circulating VEGF, produced *de novo* in the spleen, and its blockage led to protection of mice from this syndrome. In addition, either splenectomization or administration of the anti-inflammatory molecule carbon monoxide led to a significant reduction in the levels of sera VEGF and to protection from ALI. The similarities between the physiopathological lesions described here and the ones occurring in humans, as well as the demonstration that VEGF is a critical host factor in the onset of malaria-associated ALI in mice, not only offers important mechanistic insights into the processes underlying the pathology related with malaria but may also pave the way for interventional studies.

## Author Summary

Malaria remains a major source of morbidity and mortality throughout the tropical regions of the world causing up to 1 million deaths every year, mainly in children. Although infection with malaria parasites is common, only 1 to 2% of infections lead to severe life-threatening disease characterized by a range of clinical features including coma, severe anemia, respiratory distress, metabolic acidosis, or multiorgan failure. Animal models of infection are indispensable tools to better understand the dynamic host-parasite interactions that lead to the onset of different infection outcomes. We now show that DBA/2 mice infected with *P. berghei* ANKA constitute a rodent model for malaria-associated acute lung injury (ALI). Up to 60% of these infected mice develop respiratory problems including dyspnea, airway obstruction and hypoxemia and die soon after. The most common pathological findings were pleural effusion, pulmonary hemorrhage and edema, features common to human malaria patients that show life-threatening respiratory distress. Malaria-associated ALI in this model correlates with high levels of circulating vascular permeability factor, VEGF, and its blockage by different means leads to protection from ALI. The existence of such a model of disease will certainly contribute to a better understanding of malaria-associated pathology and possibly to the design of novel intervention strategies.

## Introduction

Malaria is one of the most devastating diseases in the world today. The total burden of disease has recently been estimated to be higher than 500 million episodes annually being responsible for 18% of all childhood deaths in sub-Saharan Africa, equivalent to 800,000 deaths each year. It is caused by Apicomplexan parasites of the genus *Plasmodium*, which are transmitted through the bite of a female *Anopheles* mosquito. Infection begins when an infected mosquito bites a mammalian host and deposits *Plasmodium* sporozoites under the skin. These then enter the circulatory system to reach the liver where they infect hepatocytes leading to the release of thousands of merozoites into the bloodstream, initiating the symptomatic stage of the infection (reviewed in [1], [2]).

In endemic areas, many infections in semi-immune and immune children and adults present themselves as uncomplicated febrile illness. In more severe disease, non-immune individuals may exhibit a number of syndromes including severe anemia (SA), cerebral malaria (CM) or respiratory distress (ALI/ARDS) [1]. While CM is the most studied form of severe *P. falciparum* malaria, ALI/ARDS are not only important complications in severe *P. falciparum* malaria but have been also described in *P. vivax* and *P. ovale* malaria. Malaria-associated ALI/ARDS causes high mortality and is more common in adults than in children and pregnant women, with non-immune individuals being more prone to develop this condition [3].

Malaria-associated pathogenesis is considered multi-factorial, with both host and *Plasmodium* factors playing critical roles [1], [4]. Nevertheless, the mechanisms responsible for severe malaria's high morbidity and mortality remain poorly understood [5]. This explains why no therapeutic strategies attempting to control the onset of severe malaria have been successfully developed. Laboratory mice infected with natural species of rodent malaria are indispensable tools in the search for pathways involved in the different syndromes developed during infection [6]. Here, we report on a rodent model for malaria-associated ALI. Thirty to 60% of the DBA/2 mice infected with *P. berghei* ANKA showed not only dyspnea before death but also airway obstruction, hypoxemia, pleural effusion, pulmonary hemorrhage and edema, and increased lung vessel permeability. In this model, ALI is associated with high levels of circulating VEGF and its blockade during infection led to protection of mice from this syndrome, opening new avenues to the treatment of this form of severe malaria.

## Results

### Infection of DBA/2 mice with *P. berghei* ANKA constitutes a rodent model for malaria-associated acute lung injury (ALI)

**Figure 1:**
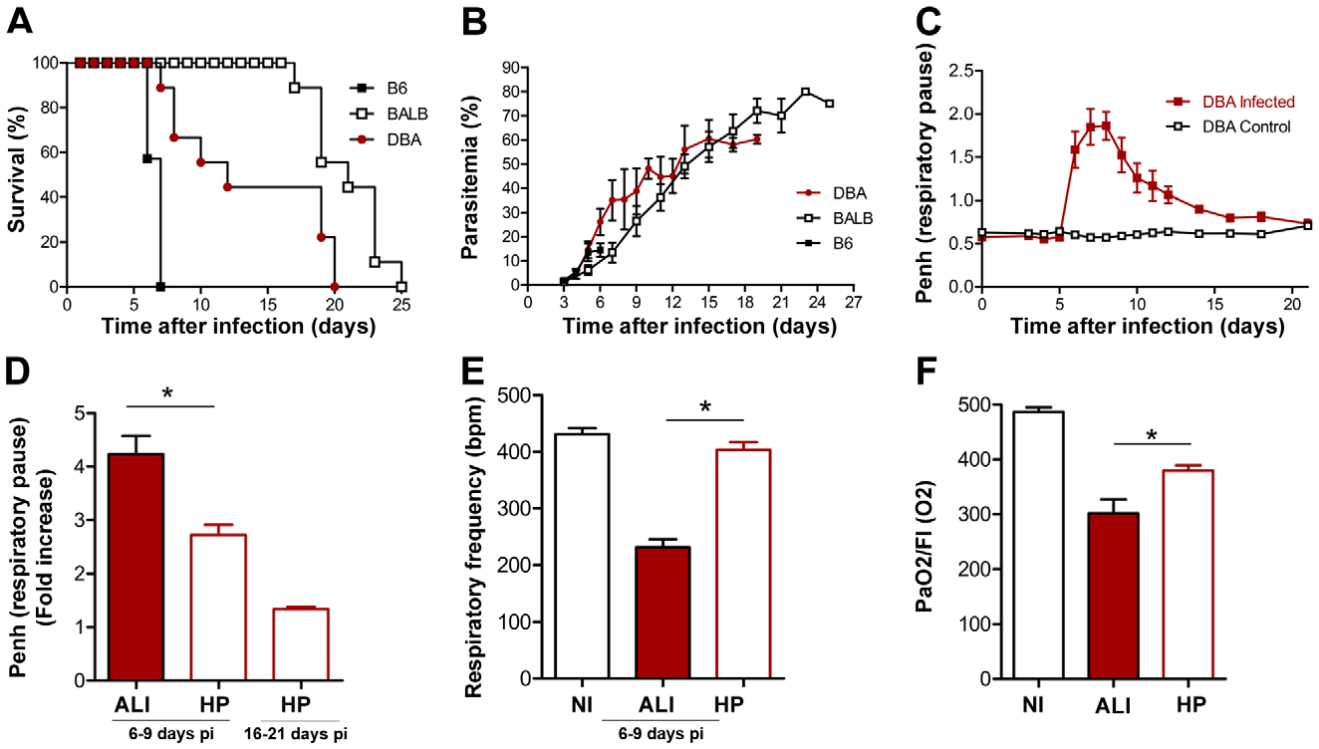
Infection of C57BL/6, BALB/c and DBA/2 mice with *P. berghei* ANKA. (**A**) Survival and (**B**) parasitemia curves are shown for C57BL/6 (B6) (n = 7), BALB/c (n = 9) and DBA mice (n = 9) and mice. Parasitemias are shown as mean ± standard deviation. (**C, D**) Penh (enhanced pause) values for non-infected (NI) *versus P. berghei* ANKA infected DBA mice (n = 21). (**E**) Respiratory frequency values for non-infected (NI) *versus P. berghei* ANKA-infected DBA mice (n = 21). ALI and HP groups were defined at the end of each experiment according to cause of death. (**F**) PaO2/FI(O2) values for non-infected (NI) *versus P. berghei* ANKA infected DBA mice (n = 6). Values for ALI mice were obtained after the onset of ALI symptoms. Values for HP mice were obtained after day 12 of infection and on mice not displaying ALI symptoms. Results are shown as mean concentration ± standard deviation. (*P<0.05).

With the aim of identifying host factors involved in the onset of distinct severe malaria syndromes, we investigated the cause of death of different mouse strains infected with the same rodent *Plasmodium* strain. Infection of 3 different mouse strains, C57BL/6, BALB/c and DBA/2 mice, with *P. berghei* ANKA-infected red blood cells (iRBCs) showed 3 significantly distinct patterns of survival curves (*P*<0.05 for C57BL/6 versus DBA/2, *P*<0.01 for DBA/2 versus BALB/c and *P*<0.001 for C57BL/6 versus BALB/c). As previously described, all C57BL/6 mice infected with *P. berghei* ANKA succumbed within 6–9 days (n = 7, **Figure 1A**) due to the development of a complex neurological syndrome consisting of hemi- or paraplegia, head deviation, tendency to roll-over on stimulation, ataxia and convulsions. Given its similarities to human CM, this neurological syndrome is referred to as experimental cerebral malaria (ECM) (*reviewed in* [7]). On the other hand, BALB/c mice are much less susceptible to developing ECM when infected with *P. berghei* ANKA. Thus, none of these mice died with ECM (n = 9) dying later (after 15 days of infection) with hyperparasitemia (HP) (>50% of infected red blood cells) (**Figure 1A**) without exhibiting any neurological symptoms. DBA/2 mice infected with *P. berghei* ANKA showed a pattern of survival distinct from the previous two strains. These mice died between days 7 and 20 after infection (**Figure 1A**). Thorough examination allowed us to discriminate two different phenotypes in *P. berghei* ANKA-infected DBA/2 mice: one that occurred in mice that died up to day 12 after infection and the other that occurred in mice that succumbed from day 12 onwards. The mice that died after 12 days of infection showed signs of severe anemia, consistent with their high levels of parasitemia (>50%, **Figure 1B**). This is similar to the HP phenotype, also observed for BALB/c mice. Importantly, none of the DBA/2 mice that died between days 7–12 after infection showed any symptoms of ECM (as observed for C57BL/6 mice). Instead, these mice showed dyspnea before death and airway obstruction, as determined by enhanced pause (Penh). These mice show significantly higher Penh values as well as lower respiratory frequency, than non-infected and *P. berghei* ANKA-infected DBA/2 mice that died later with HP (**Figure 1C, D, E**). Importantly, these mice are hypoxemic after the onset of the symptoms, with PaO_2_/fraction of inspired oxygen (FIO_2_) values below 300 mmHg and significantly lower than non-infected and *P. berghei* ANKA-infected DBA/2 mice without symptoms (*P*<0.001; **Figure 1F**). *Post-mortem* studies revealed that the main pulmonary necroscopic finding observed in 100% of these mice was pleural effusion. Analysis of the pleural fluid from these mice (n = 10) revealed to be an exsudate (high total protein content, 59.4±11.7 mg/ml, showing specific-gravity >1.020, 1.030±0.004) that contained inflammatory cells such as neutrophils (57.6±11.7%), lymphocytes (28.5±15.1%), monocytes and macrophages (13.8±6.8%), as well as both infected and non-infected red blood cells.

ALI and ARDS are both disorders of the lung with similar features to those described above for *P. berghei* ANKA infected DBA mice, such as dyspnea and respiratory insufficiency (as first symptoms) as well as inflammatory infiltrates and hypoxemia. Importantly, ALI and ARDS differ only in the degree of hypoxemia, defined as PaO_2_/FiO_2_ ≤300 mmHg (for ALI) or ≤200 mmHg (for ARDS). Thus, *P. berghei* ANKA infected DBA/2 mice, which show all these features including hypoxemia with PaO_2_/fraction of inspired oxygen (FIO_2_) values between 200 and 300 mmHg, represent a model of malaria-associated ALI. Importantly, we also noted that none of these features were observed in DBA/2 mice infected with other *Plasmodium* strains, including *P. berghei* NK65, *P. chabaudi chabaudi* AS and *P. yoelii yoelii* 17X (data not shown) suggesting that the onset of malaria-associated ALI in mice depends on the specific *P. berghei* ANKA-DBA/2 combination.

**Figure 2:**
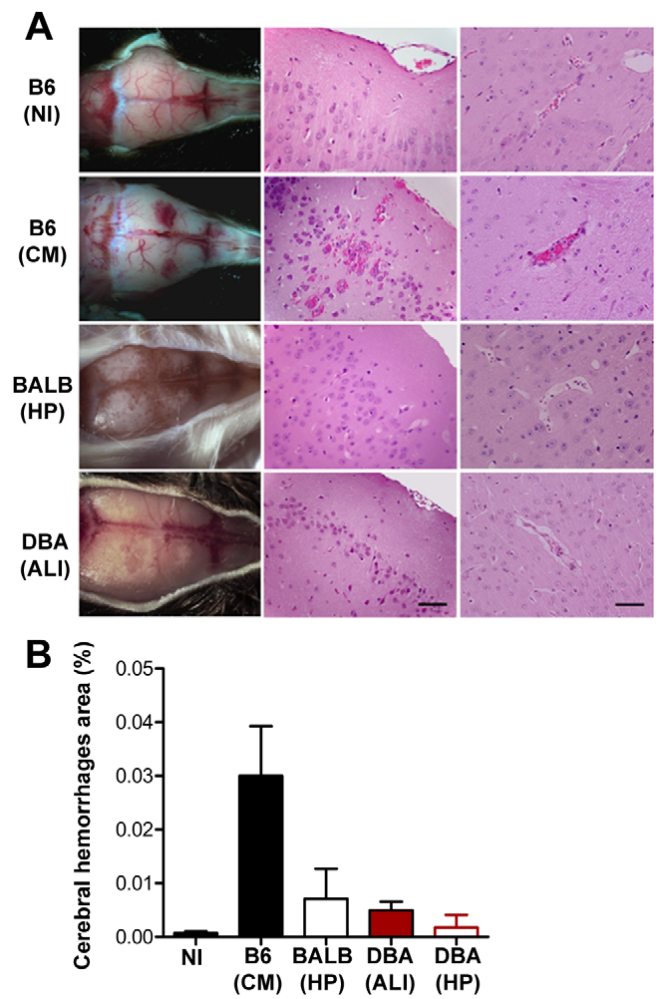
Infection of DBA/2 with *P. berghei* ANKA does not cause brain damage. (**A**) Cranium and Hematoxylin-Eosin staining of brain sections of *P. berghei* ANKA-infected C57BL/6, B6 (CM), BALB/c, BALB (HP), and DBA mice, DBA (ALI) or DBA (HP) (5 µm). Images are representative of 9 mice in 3 independent experiments. (**B**) Quantification of cerebral hemorrhagic foci area in brain-sections of the same group of mice. Results are shown as mean concentration ± standard deviation (n = 9 animals per group).

Given that these mice die within a similar time scale as C57BL/6 mice infected with *P. berghei* ANKA, we sought to determine the main differences between the ECM and malaria-associated ALI syndromes. The main CNS (central nervous system) necroscopic and histological findings in *P. berghei* ANKA-infected C57BL/6 mice were hemorrhages in the cranium, brain and cerebellum (**Figure 2A**). Histopathological examination also showed multifocal hemorrhages in white and grey matters (pyramidal, molecular and granular layers, perivascular, hippocampus, and bulb) and congestive blood vessels in 100% of the C57BL/6 mice showing ECM symptoms (**Figure 2A, B**). However, only 20% of the *P. berghei* ANKA-infected DBA/2 mice with ALI symptoms showed some hemorrhagic foci (*data not shown*), which were much smaller and less frequent than the ones observed in *P. berghei* ANKA-infected C57BL/6 mice. In addition, none of the mice show ECM symptoms (**Figure 2B**).

**Figure 3:**
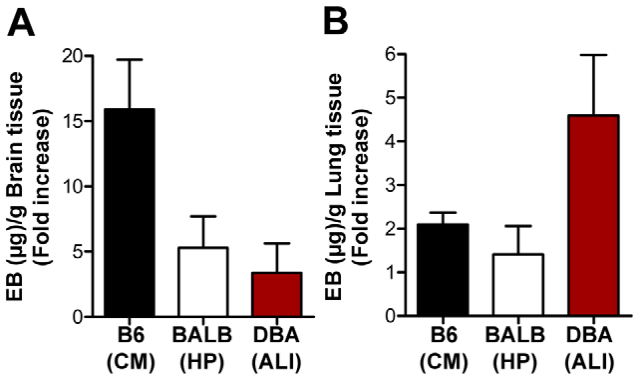
Infection of DBA/2 with *P. berghei* ANKA leads to lung vascular permeabilization without compromising BBB. (**A**, **B**) Assessment of BBB and lung vascular permeabilization by Evans Blue (EB) quantification, during the onset of ECM, i.e. C57BL/6 mice, B6 (CM), hyperparasitemia, i.e. BALB/c mice, BALB (HP), and ALI, i.e. DBA mice, DBA (ALI). Evans Blue quantification is shown as mean µg of Evans Blue (EB) per g of brain or lung tissue ± standard deviation (*n = *5–11 animals per group).

**Figure 4:**
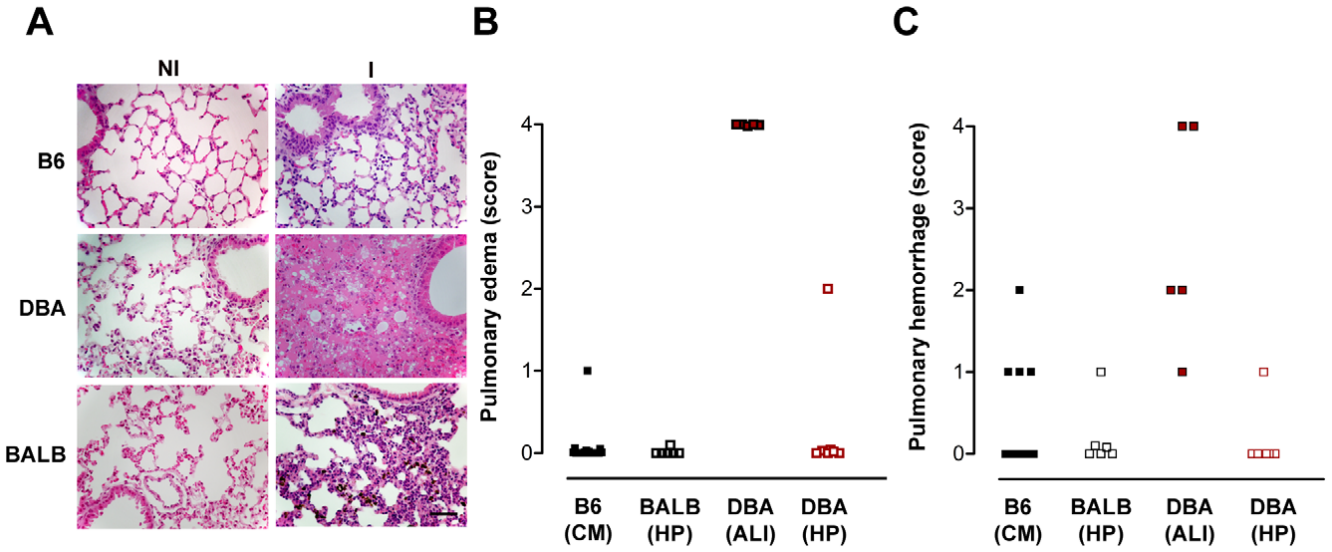
Infection of DBA/2 with *P. berghei* ANKA constitutes a rodent model for malaria-associated acute lung injury (ALI). (**A–C**) Hematoxylin-Eosin staining of lung sections (5 µm) and quantification of edema and haemorrhages, using a blinded scoring system (1-mild, 4-severe), during the onset of ECM, i.e. C57BL/6 mice, B6 (CM), hyperparasitemia, i.e. BALB/c mice, BALB (HP), and ALI, i.e. DBA mice, DBA (ALI). Images are representative of 9 mice in 3 independent experiments. The bar corresponds to 100 µm.

A hallmark of ECM is the disruption of the blood-brain barrier (BBB). Indeed, 100% of *P. berghei* ANKA-infected C57BL/6 presented, during the onset of ECM, disruption of the BBB, as revealed by a ∼15-fold increase in Evans blue accumulation in brain parenchyma, as compared with non-infected C57BL/6 controls (**Figure 3A, ***P*<0.0001). In contrast, BBB disruption was not observed in *P. berghei* ANKA-infected DBA/2 or BALB/c mice (**Figure 3A**). Instead, lung vessel permeability was significantly higher in infected DBA/2 mice showing ALI symptoms (*P*<0.001) but not in C57BL/6 or BALB/c mice (**Figure 3B**). Pulmonary edema has been correlated with impaired gas exchange within the lungs, ultimately leading to severe respiratory failure and death [8]. This condition can originate from a number of insults involving damage to the alveoli capillary membrane, including direct pulmonary injury (*e.g.*, pulmonary infection) and indirect injury (*e.g.*, sepsis) [8]. Indeed, while severe pulmonary edema and hemorrhages were observed in 100% of the *P. berghei* ANKA-infected DBA/2 mice showing ALI symptoms, this was a rare event in mice dying with ECM and was never extensive or severe enough to constitute the cause of death. Altogether, these data show that the two experimental syndromes are distinct. While in ECM the brain is the major affected organ, the lung is the key organ in the onset of ALI in *P. berghei* ANKA-infected DBA/2 mice. Major histopathological changes in the lungs of DBA/2 mice after the onset of ALI are characterized by inflammatory cellular infiltration (neutrophil-dominant and foamy macrophages in the alveolar and interstitial sites) as well as marked alveolar edema and hemorrhage. It is interesting to note that both DBA/2 and BALB/c mice showing HP also show interstitial pneumonia but the histopathological features were very distinct from those of DBA/2 mice with ALI. Mice with HP showed a thickened alveolar septum with some mononuclear inflammatory cells. The lung pattern observed in C57BL/6 after the onset of ECM was characterized by a discrete presence of mononuclear inflammatory cells and/or polymorphonuclear leucocytes but, in most cases without thickening of the alveolar septum (**Figure 4A, B**).

### VEGF is a critical host factor for the onset of malaria-associated ALI

**Figure 5:**
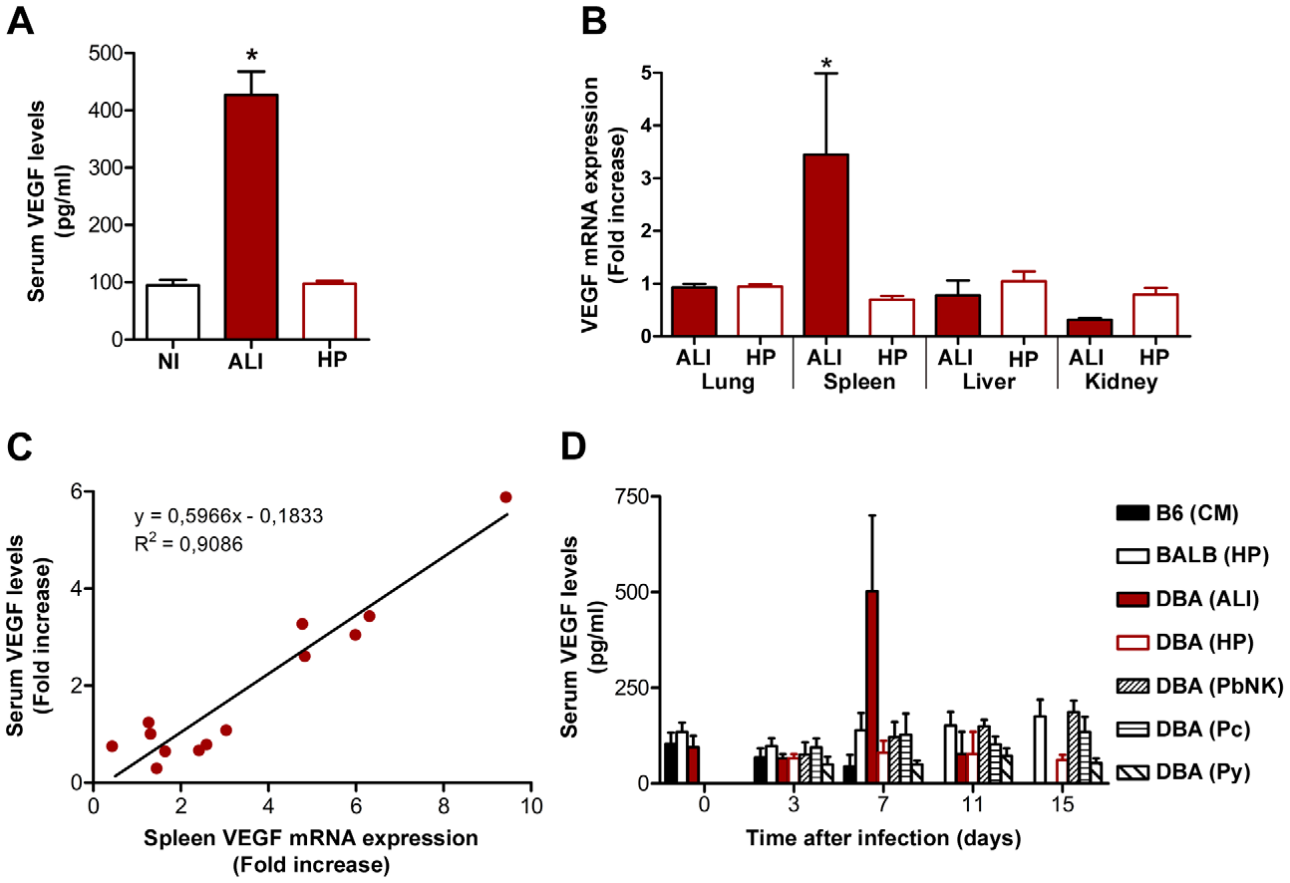
VEGF correlates with ALI onset during malaria infection. (**A**) Levels of VEGF protein in the sera of *P. berghei* ANKA-infected DBA mice with ALI and hyperparasitemia (HP), compared to non-infected mice (NI). Results are shown as mean concentration ± standard deviation (n = 6, 23 and 46 mouse sera per group, for NI, ALI and HP, respectively). (**B**) Expression of VEGF mRNA levels in the lung, spleen, liver and kidney of *P. berghei* ANKA-infected DBA mice with ALI and HP (n = 15 animals per group), when compared to non-infected mice. (**C**) Correlation between VEGF protein levels in the serum and mRNA expression of VEGF in the spleen of *P. berghei* ANKA-infected DBA mice with ALI. (**D**) Levels of VEGF protein in the sera of different strains of mice (C57BL/6, BALB/c and DBA) infected with different *Plasmodia* (*P. berghei* ANKA, *P. berghei* NK65 - PbNK, *P. yoelii yoelii* 17X – Py, and *P. chabaudi chabaudi* AS - Pc) (n = 10 animals per group). Results are shown as mean concentration ± standard deviation. (*P<0.001).

Our next objective was to determine the cause of the development of malaria-associated ALI, using *P. berghei* ANKA-infected DBA/2 mice as a model. Pulmonary edema, i.e., fluid build-up in the lung alveoli, originates in the loss of the integrity of the alveolar-capillary barrier (**Figure 3B**). Indeed, increased alveolar permeability is considered to be the key functional abnormality underlying malaria-associated ALI/ARDS in humans, as seen in ALI/ARDS due to other causes. However, the mechanisms underlying the onset of this syndrome in humans are still not known. It has been shown that vascular endothelial growth factor, VEGF, plays a critical role in angiogenesis but also in vascular permeability [9]. Indeed, systemic overexpression of VEGF has been shown to cause widespread capillary leakage in multiple organs, especially in the lungs [10]. Moreover, high levels of VEGF in plasma were found in ARDS patients [11]. To address the role of VEGF in malaria-associated ALI, VEGF levels in the sera of *P. berghei* ANKA-infected DBA/2 mice were measured throughout infection. VEGF levels remained constant throughout infection, except for the mice that developed ALI, which showed a significant increase in VEGF levels by day 7 after infection (**Figure 5A**). The systemic increase in VEGF in the sera seems to originate from *de novo* production in the spleen (**Figure 5B**), as shown by the correlation between mRNA levels in the spleen and serum protein levels (**Figure 5C**). No major alterations of VEGF levels were observed in DBA/2 mice infected with *P. berghei* NK65, *P. c. chabaudi* AS or *P. yoelii yoelii* 17X, similarly to C57BL/6 and BALB/c mice infected with *P. berghei* ANKA, none of which developed ALI symptoms (**Figure 5D**). Altogether, these data show that high VEGF levels in plasma correlate with the onset of ALI in malaria-infected mice. VEGF is known to cause an increase in lung vascular permeability, which strongly supports the idea that higher levels of VEGF in serum might be the cause of malaria-associated ALI.

**Figure 6:**
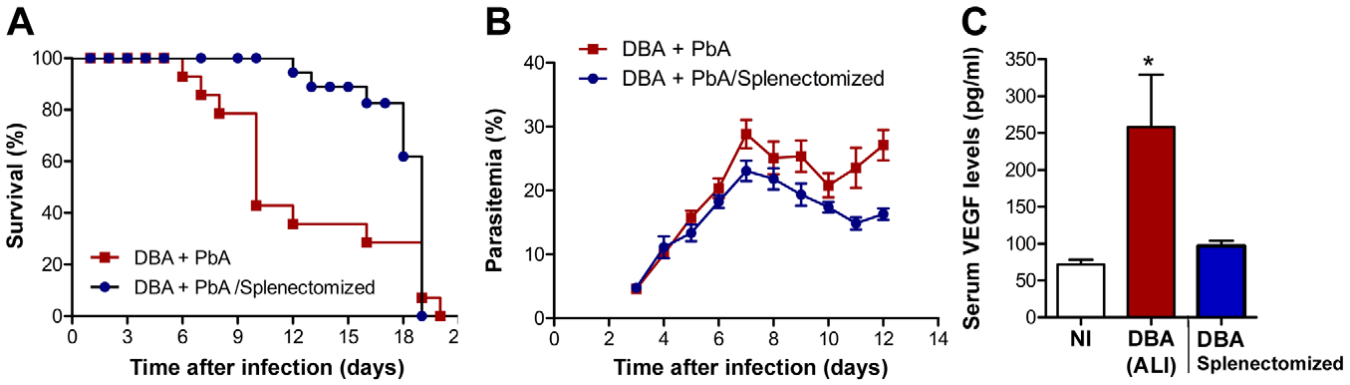
Spleen is required for the onset of malaria-associated ALI. (**A**) Survival and (**B**) parasitemia of splenectomised and control *P. berghei* ANKA-infected DBA mice. (**C**) Levels of VEGF in the serum of non-infected (NI) DBA mice, *P. berghei* ANKA-infected DBA mice with ALI and splenectomised *P. berghei* ANKA-infected DBA mice were taken on the same day as control DBA infected mice developed ALI. Results are shown as mean concentration ± standard deviation. (*P<0.01).

These results strongly suggest that increased levels of VEGF in circulation originate from *de novo* production in the spleen and may be the cause of death in *P. berghei* ANKA-infected DBA/2 mice that develop ALI. Thus, we next asked whether *P. berghei* ANKA-infected splenectomized DBA/2 mice would be protected from developing ALI. The results clearly show that the spleen is required for the onset of malaria-associated ALI, which correlates with VEGF levels in circulation (**Figura 6A–C**).

**Figure 7:**
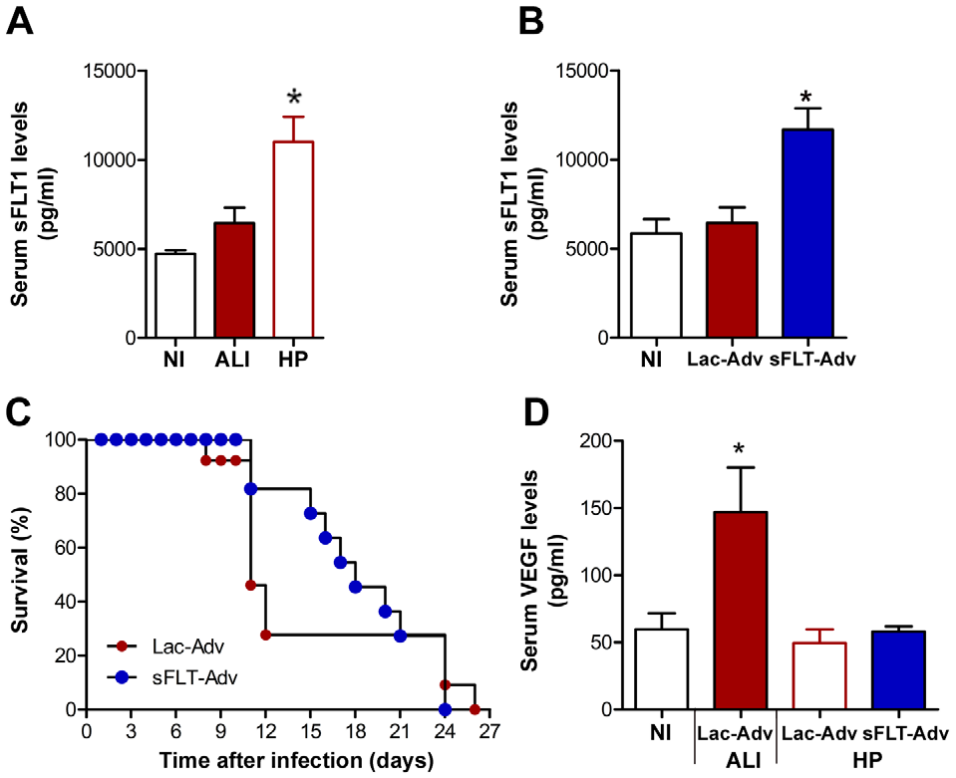
VEGF promotes ALI onset in mice with malaria. (**A**) Levels of the soluble form of VEGF receptor (sFLT1) in the serum of *P. berghei* ANKA-infected DBA mice with ALI or mice in HP group. Since only small volumes of blood from the mouse tail vein can be used for this determination, the group classification was only performed by the end of each experiment by determining the cause of death. (**B**) Levels of the soluble form of VEGF receptor (sFLT1) in the serum of *P. berghei* ANKA-infected DBA mice after the administration, by intraperitoneal injection on day 3 and day 5 after infection, of LacZ and sFLT1-expressing adenoviruses (n = 11 animals per group). Results are shown as mean concentration ± standard deviation. (**C**) Survival of LacZ and sFLT1-expressing adenoviruses treated *P. berghei* ANKA-infected DBA mice. (**D**) Levels of VEGF in the serum of the control LacZ-expressing adenoviruses-treated *P. berghei* ANKA-infected DBA (ALI and non-ALI/HP) mice *versus* the sFLT1-expressing adenoviruses-treated *P. berghei* ANKA-infected DBA (non-ALI/HP) mice. Results are shown as mean concentration ± standard deviation. (*P<0.05).

Infected DBA/2 mice that did not develop ALI not only showed unaltered levels of VEGF in the sera (**Figure 5A**), but also showed a significant increase in the levels of the soluble form of the VEGF receptor (sFLT1) (**Figure 7A**), known to neutralize excess VEGF in circulation [12], [13], [14], [15]. Therefore, it is reasonable to think that interfering *in vivo* with VEGF levels might protect mice from the onset of malaria-associated ALI. To this end, sFLT1-expressing adenoviruses were administered intravenously (i.v.) into DBA/2 mice on days 3 and 5 after infection with *P. berghei* ANKA. LacZ-expressing adenoviruses were administered to control mice. Administration of sFLT1-expressing adenoviruses led to a significant increase of sFLT1 expression (**Figure 7B**, 1.8 fold, *P*<0.05). While in the control group approximately 70% (n = 8 mice out of 11) of the mice died with malaria-associated ALI symptoms, only 18% (n = 2 mice out of 11) of the mice treated with sFLT1-expressing adenoviruses succumbed to this syndrome (**Figure 7C**). The protection from malaria-associated ALI fully correlated with a significant decrease in VEGF levels in circulation (66% decrease between mice developing malaria-associated ALI and non-ALI in the group receiving LacZ-adenoviruses and 63% decrease between malaria-associated ALI-developing mice receiving LacZ-adenoviruses and non-ALI mice receiving sFLT1-adenoviruses, *P*<0.05 or *P*<0.005, respectively) (**Figure 7D**). Altogether, these data demonstrate that VEGF is a critical host factor for the onset of malaria-associated ALI in mice.

### Administration of a potent anti-inflammatory molecule by inhalation suppresses the onset of malaria-associated ALI

**Figure 8:**
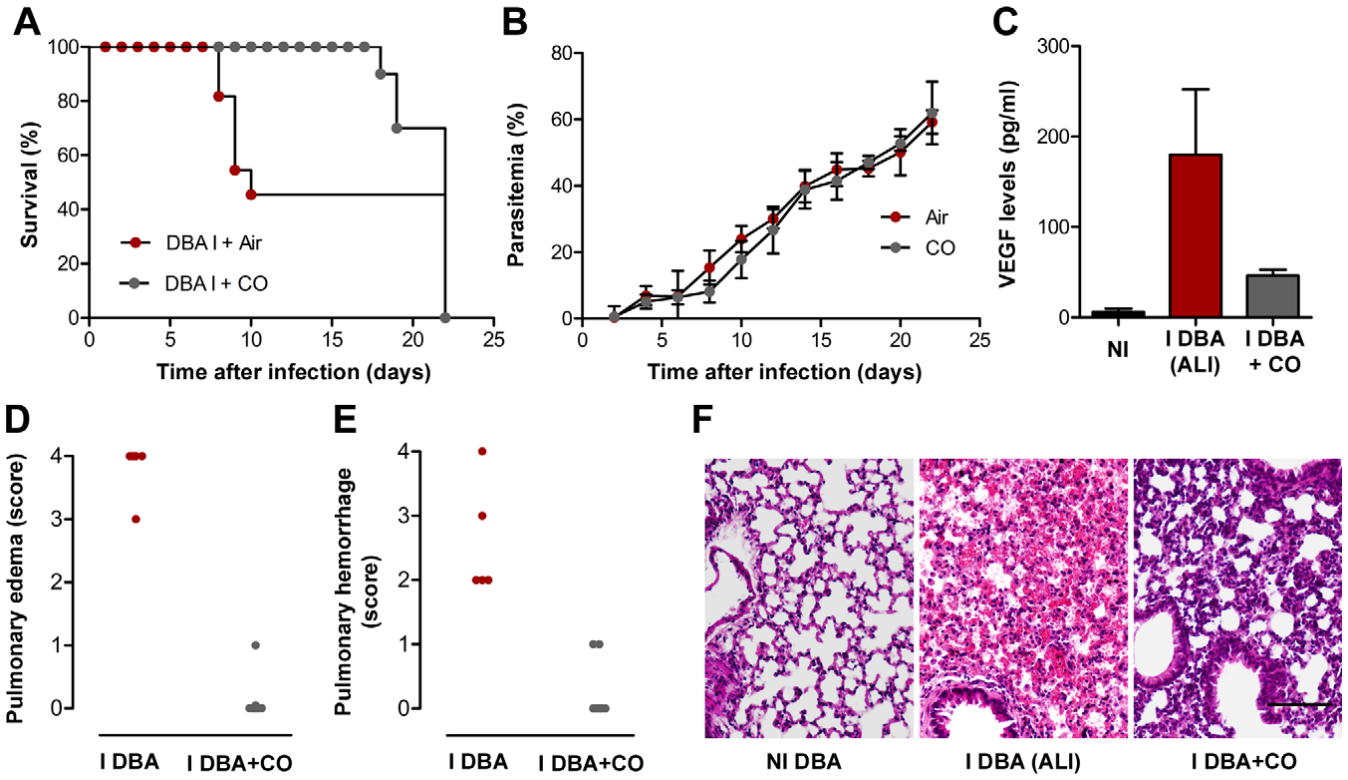
Exposure to CO suppresses onset of malaria-associated ALI. (**A–B**) Effect of CO inhalation on *P. berghei* ANKA-infected DBA mice survival (**A**) and parasitemia (**B**). CO inhalation (starting at day 2 after infection and during 72 h) was compared to normal atmosphere. Parasitemias are shown as mean ± standard deviation (*n = *10 animals per group). (**C**) Levels of VEGF in the sera of *P. berghei* ANKA-infected DBA mice exposed to air or CO. Results are shown as mean concentration ± standard deviation (n = 3 mice per group). (*P<0.01) (**D–F**) Quantification of edema and haemorrhages of hematoxylin-eosin-stained lung sections of non-infected DBA mice (NI DBA) *versus P. berghei* ANKA-infected DBA mice exposed to air, I DBA (ALI), or CO, I DBA+CO, using a blinded score system (1-mild, 4-severe). Images are representative of 6 mice in 2 independent experiments.

Despite the distinct outcomes observed, the host inflammatory response has been postulated to play a major role in the onset of distinct severe forms of malaria infection [16]. In the case of *P. berghei* ANKA-infected DBA/2 mice, it is also tempting to speculate that an uncontrolled inflammatory response of the host to the parasite might be the primary cause of the observed VEGF increase. This hypothesis is strongly supported not only by the presence of inflammatory cells in the pleural exsudate but also by the fact that the spleen is the major contributor to VEGF increase. We have previously shown that administration of a potent anti-inflammatory molecule, carbon monoxide (CO), suppresses the pathogenesis of ECM [17], [18]. Interestingly, a similar administration of exogenous CO has been shown to be beneficial on a number of lung injury models (reviewed in [19]). When CO (250 parts per million; p.p.m.) was administered for 72 h, starting at day 2 after infection, it prevented death of *P. berghei* ANKA-infected DBA/2 mice by ALI (**Figure 8A**) without significant alterations in the parasitemia (**Figure 8B**) but with a significant impairment in the increase on the levels of VEGF in circulation (*P*<0.01; **Figure 8B**). Moreover, our histopathological observations showed that lungs from mice under CO administration did not present hemorrhages and pulmonary edema (**Figure 8D–F**). These data not only reveal a means of preventing the onset of malaria-associated ALI but also strongly suggest that, as for ECM, the host inflammatory response may play an important role in the onset of this severe malaria syndrome.

## Discussion

Once thought to be near eradication, malaria is now one of the most prevalent infectious diseases worldwide, with a toll of nearly 1 million deaths per year in regions where infection is endemic. These deaths are at the most severe end of a scale of pathologies affecting approximately 500 million people per year and can be due to the onset of distinct syndromes. These include cerebral malaria (CM), acute lung injury (ALI), acute respiratory distress syndrome (ARDS), and severe anemia, among other pathologies. The outcome of infection is influenced by the genetics of both host and parasite [4], [20]. This is particularly visible in rodent models of infection, as different strains of mice infected with different *Plasmodium* strains develop a variety of pathologies, ranging from lethal to self-resolving [18], [21]. Rodent models that mimic certain aspects of the human CM, anemia syndromes and pregnancy-associated malaria have been established [6], [22], [23]. We now report that DBA/2 mice infected with *P. berghei* ANKA constitute a model for malaria-associated ALI, where the cause of death is respiratory failure. It is important to note that *P. berghei* ANKA-infected DBA/2 mice have been previously described as CM-resistant [24]. However, another report has described these mice as a resolving CM model. Interestingly, the authors also noted changes in vascular permeability in DBA/2 mice during what they called “mild cerebral malaria” phase. They further state that the even distribution of these changes suggests a response to a circulating factor, although they do not speculate on which factor that might be [25]. Our present detailed pathological study, of the brain and the lungs of *P. berghei* ANKA- infected DBA/2 mice, indicates that the cause of death of these mice is respiratory failure.

In humans, while patients with uncomplicated malaria usually present fever and non-specific symptoms, severe and complicated malaria is characterized by multiorgan involvement including ALI/ARDS. Recent years have witnessed a shift in the profile of patients with complicated malaria (reviewed in [3]). Multi-organ system failure and respiratory complications are being increasingly reported not only for *P. falciparum* infections but also for malaria caused by *P. vivax* [26], [27], [28], [29], *P. ovale* [30] and *P. malariae* [31], usually considered benign *Plasmodium* species. In fact, it has been suggested that as many as 5% of patients with uncomplicated malaria and 20–30% of patients with severe and complicated malaria requiring intensive care unit admission may develop ALI/ARDS, often after treatment has been initiated [3]. Pregnant women with severe *P. falciparum* infection are particularly prone to developing ALI/ARDS, which is associated with high mortality [32], [33]. It is therefore of the utmost importance that a rodent model of such syndrome becomes available. Moreover, *post-mortem* studies on human patients dying with severe *P. falciparum* malaria have revealed histopathological findings, such as heavy edematous lungs and hemorrhages [34], [35], very similar to the ones we describe here for *P. berghei* ANKA-infected DBA/2 mice developing malaria-associated ALI. Mild lung pathology has been previously reported in C57BL/6 mice infected with *P. berghei* ANKA [36], [37]. Our present study confirms that C57BL/6 mice died with a significant loss of the integrity of the BBB, causing all the ECM symptoms observed prior to death, but also showed some level of lung pathology. However, none of those mice presented pleural effusion or exsudate in their pleural cavities. Moreover, while pulmonary edema and hemorrhages were observed in 100% of the *P. berghei* ANKA-infected DBA/2 mice showing ALI symptoms, this was a rare event in *P. berghei* ANKA-infected C57BL/6 mice and was never severe enough to constitute the cause of death. *Plasmodium* blood stage infection is known to cause multi-organ pathology but the level of pathology varies from organ to organ depending on the host-*Plasmodium* combination. Here, we clearly show that infection of C57BL/6 or DBA/2 mice with *P. berghei* ANKA results into two distinct models of severe malaria; the former developing a neurological syndrome while the latter causing death due to respiratory failure in approximately half of the infected mice.

Importantly, our data also show that a host factor plays a critical role in the establishment of malaria-associated ALI. Indeed, the present data demonstrates that *P. berghei* ANKA only causes malaria-associated ALI in DBA/2 mice. Interestingly, DBA/2 mice have been shown to respond quite strongly to angiogenic stimuli [38] and this might be the reason why a proportion of these mice are not able to control the levels of VEGF, leading to the onset of ALI during a *P. berghei* ANKA infection. It should also be noted that a model named “malaria lung syndrome”, where C3H/z mice infected with *P. berghei* K173 also die very early in infection and show notably edematous lungs and pleural effusion, has been described more than 25 years ago [39]. Although it would be very interesting to test the levels of VEGF in these mice, the unavailability of this strain of mice from the major animal houses makes this experiment very difficult to perform.

But why is VEGF responsible for the onset of malaria-associated ALI? VEGF has long been known for its activity as a regulator of vessel permeability [13]. In fact VEGF was primarily termed vascular permeability factor, for its ability to induce vascular leakage, rather than for its growth factor activity [40]. VEGF increases vascular permeability 50,000 times more efficiently than does histamine [41]. Interestingly, VEGF also plays a central role in the formation and maintenance of lung vasculature [42]. However, when VEGF levels are altered, lung disease frequently follows. Plasma VEGF levels in subjects with non-malaria ALI/ARDS are strongly elevated compared to controls and values higher than two-fold have been associated with mortality [11]. The association between VEGF levels and mortality due to respiratory failure does not mean that VEGF effects are restricted to the lung, but simply highlights the importance of vascular integrity for lung function. Another example in which VEGF and lung injury are involved in response to a pathogenic microorganism has recently been reported [43]. *Pseudomonas aeruginosa* is a pathogenic bacterium that colonizes the lungs and may lead to lung disease in immunocompromized patients. Interestingly, while aerosol delivery of this bacterium causes fatal disease in DBA/2 mice, other mouse strains are able to resolve infection. DBA/2 mice display progressive deterioration of lung pathology with extensive alveolar exsudate and edema formation together with significantly increase levels of VEGF that seem to result from an uncontrolled host inflammatory response [43]. Indeed, a cross-talk between angiogenesis and inflammation has long been proposed [44]. Similarly, *P. berghei* ANKA-infected DBA/2 mice treated with a potent anti-inflammatory molecule prior to the onset of ALI show significantly reduced levels of VEGF in sera and are fully protected from this syndrome of severe malaria.

Numerous studies have measured VEGF levels in malaria patients [45], [46], [47] but none of these studies included a group of individuals for which the cause of death was ALI/ARDS. On the other hand, it was recently shown that *P. falciparum*-infected red blood cells induce VEGF secretion from human mast cells, a cell population highly represented in the spleen [48]. Importantly, while ALI affects pregnant women infected with *P. falciparum* [32], the VEGF pathway seems to play an important role during chronic placental malaria and hypertension in first-time mothers [49]. It remains to be established whether these observations are in any way connected. The similarities between the physiopathological lesions described in the rodent model reported here and those occurring in humans pave the way for a better understanding of the malaria-associated pathology and may contribute to the design of novel rational intervention strategies.

## Methods

### Mice

C57BL/6, BALB/c and DBA-2 mice were bred and housed in the specific pathogen-free facilities of the Instituto de Gulbenkian de Ciência. The mice were then transferred to the Instituto de Medicina Molecular at least 72 h prior to experimentation. All protocols were approved by the Animal Care Committee of the Instituto de Medicina Molecular, following Institutional, National, and European Union guidelines.

### Parasites, infection and disease assessment

*P. berghei* ANKA, *P. berghei* NK65, *P. yoelii* 17X or *P. chabaudi* AS were used after one *in vivo* passage in C57BL/6, BALB/c or DBA-2 mice. Mice were infected via intraperitoneal (ip) inoculation with 10^6^–10^7^ infected red blood cells. Infected mice were monitored twice daily for clinical symptoms of ECM including hemi- or paraplegia, head deviation, tendency to roll-over on stimulation, ataxia and convulsions or ALI, including dyspnea. Parasitemia was determined by Giemsa staining followed by microscopic counting and expressed as percentage of infected red blood cells.

### Histopathology

Brains or lungs were harvested from mice under different experimental conditions when clinical signs of ECM, ALI or HP were noticed. Tissues were fixed in buffered 10% (v/v) formaldehyde for paraffin embedding and Hematoxylin-Eosin staining.

### Determination of airway obstruction

Pulmonary function was assessed in unrestrained conscious mice placed in a barometric plethysmographic chamber (Buxco Electronics, Sharon, CT), where respiratory parameters were measured every day for 10 minutes. Since these measurements can be performed every day in the same group of mice, the group classification was only performed by the end of each experiment after determining the cause of death. The enhanced pause (Penh), a dimensionless value indicative of airway obstruction, as well as respiratory frequency, were used to determine respiratory resistance and were calculated as previously described [50].

### Measurement of PaO_2_ in arterial blood

Mice were gently heated in their cages with a heat lamp to increase peripheral blood flow. The mice were then restrained in a restraining device, and the ventral artery of the tail was nicked by carefully plunging a small scalpel blade diagonally into the artery. Heparin was swabbed onto the skin before it was cut to minimize clotting. About 100 mL of blood was collected in a lithium-heparin (50 IU/ml) containing capillary tube Blood in the capillary tube was mixed by placing a small metal fragment into the tube and then passing a magnet along the length of the tube several times. The samples were analyzed immediately with i-STAT cartridge CG8+ (pH, PCO2, PO2, Na, K, iCA, Glu, Hct) using the i-STAT® System Analyzer (Abbott Laboratories).

### BBB and lung permeability

Mice were injected intravenously (iv) with 0.2 ml of 1–2% Evans Blue (Sigma) when clinical symptoms of ECM, ALI or HP were noticed. Mice were sacrificed one hour later and brains or lungs were weighted and placed in formamide (2 ml) (Merck) (37°C, 48 h) to extract Evans Blue dye from the tissue. Absorbance was measured at λ = 620 nm (Bio Rad SmartSpec 3000). Evans Blue concentration was calculated from a standard curve and is expressed as µg of Evans Blue per g of brain or lung tissue.

### CO exposure

Mice were placed in a gastight 60 L capacity chamber and exposed to CO for the times indicated, as described elsewhere [18]. Briefly, 1% CO (Aga Linde) was mixed with air in a stainless steel cylinder to obtain a final concentration of 250 ppm. CO was provided continuously at a flow rate of ∼12 L/min. CO concentration was monitored using a CO analyzer (Interscan Corporation, Chatsworth). Controls were maintained in a similar chamber without CO.

### Protein levels determination

Mouse VEGF and sFLT1 levels in plasma or serum samples were determined using a commercial ELISA kit (R&D Systems) following the manufacturer's instructions. Once again, and since only small volumes of blood from the mouse tail vein can be used for this determination, the group classification was only performed by the end of each experiment after determining the cause of death.

### Quantitative RT-PCR

Extraction of total RNA from lungs, spleen, liver and kidney, from mice with ALI and HP symptoms, was performed using RNeasy Mini Kit (Qiagen), according to the manufacturer's instructions. Non-infected mice were used as controls and as baseline levels. After extraction, RNA concentration and quality were determined using a NanoDrop ND-100 spectrophotometer (NanoDrop Technologies). One microgram of total RNA was reverse-transcribed to single-strand cDNA using the AMV Reverse Transcriptase protocol (Roche Applied Science). VEGF transcripts in the cDNA pool obtained from the reverse transcriptase reaction were quantified by real-time quantitative fluorogenic PCR. SYBR Green PCR Master Mix (Applied Biosystems) was used to quantify gene expression according to the manufacturer's instructions.

RNA expression levels were calculated using the ABIPrism 7000 SDS Software, and normalized against the expression levels of the housekeeping gene hypoxanthine guanine phosphoribosyltransferase (HPRT).

### Adenovirus production

An adenoviral vector carrying the sFLT1 gene was produced using the same LR Clonase II enzyme recombination reaction as described above, but using the pAd/CMV/V5-DEST Gateway vector (Ad; Invitrogen) as destination vector. Once the sFLT1-containing Ad vector was established, an adenoviral stock was produced. A vector containing the *LacZ* gene was used as a control. After purification from the enzymatic reaction, the *Pac* I-digested vectors were transfected into 293A cells, with Lipofectamine 2000 (Invitrogen) as the transfection reagent in Opti-MEM I Medium (Gibco/Invitrogen) without serum. Cells were incubated overnight in a 5% CO_2_ incubator at 37°C. Media were replaced the following day with complete medium (DMEM with 10% Foetal Calf Serum, 2 mM glutamine, 0.1 mM non essential aminoacids and 100 U/mL penicillin, 0.1 mg/mL streptomycin). Forty-eight hours post-transfection, cells were trypsinized and transfered to sterile 10 cm tissue culture plates containing 10 mL complete medium. Media were replaced every other day until day 8, when visible regions of cytopathic effect (CPE) were observed. Infection was allowed to proceed for an additional 2 days until ∼80% CPE was observed. Adenovirus-containing cells were harvested by squirting cells off the plate with a pipette. A crude viral lysate was prepared by 3 consecutive freeze-thaw cycles (30 minutes at −80°C, followed by 15 minutes at 37°C). This crude lysate was further amplified by infection of 293A cells. After 3 days, amplified viral stocks were obtained using the freeze-thaw procedure described before. Amplified adenoviral stocks were titered using 293A cells and stored at − 80°C until use.

### Statistical analysis

For samples in which n>5, statistical analysis were performed using unpaired Student *t* or ANOVA parametric tests. Normal distributions were confirmed using the Kolmogorov-Smirnov test. For samples in which n<5, statistical analysis were performed using Kruskall-Wallis or Wilcoxon non-parametric tests. All survival curves were compared using Student *t*, Mann-Whitney e Kolmogorov-Smirnov tests. *P<*0.05 was considered significant.
